# The relationship between the use of metformin and the risk of pancreatic cancer in patients with diabetes: a systematic review and meta-analysis

**DOI:** 10.1186/s12876-023-02671-0

**Published:** 2023-02-24

**Authors:** Jian Hu, Hong-Dan Fan, Jian-Ping Gong, Qing-Song Mao

**Affiliations:** 1 Department of Hepatobiliary Surgery, The Second Affiliated Hospital of Chongqing Medical University, Chongqing, 400000 China; 2 Department of Hepatobiliary Surgery, Dianjiang People’s Hospital of Chongqing, Chongqing, 408300 China

**Keywords:** Metformin, Pancreatic cancer, Diabetes mellitus, Meta-analysis

## Abstract

**Objective:**

We aim to evaluate the relationship between the use of metformin and the risk of pancreatic cancer in type 2 diabetes patients.

**Method:**

We systematically searched the observational studies on PubMed, Embase, Web of Science, Cochrane Library, clinicalrials.gov, and CNKI databases, extracted relevant data, combined the OR value and 95% CI using the random effect model, and conducted a sensitivity analysis, subgroup analysis, and meta-regression to evaluate the size and stability of this relationship.

**Result:**

Twenty-nine studies from twenty-four articles met our inclusion criteria, including more than 2 million subjects. Overall analysis showed that compared with no use of metformin, the use of metformin could reduce the risk of pancreatic cancer in patients with type 2 diabetes (OR = 0.82, 95% CI (0.69, 0.98)). Subgroup analysis showed that compared with the use of hypoglycemic drugs, the use of metformin could reduce the risk of pancreatic cancer in patients with type 2 diabetes (OR = 0.79, 95% CI (0.66, 0.94)). However, compared with no drugs or only diet therapy, metformin users might increase the risk of pancreatic cancer (OR = 2.19, 95% CI (1.08, 4.44)). Sensitivity analysis confirmed the stability of the study, and there was no significant publication bias.

**Conclusion:**

Compared with the no-use of metformin, metformin users with diabetes can reduce the risk of pancreatic cancer. More research is needed to prove it works.

## Background

According to GLOBOCAN 2020 statistics, pancreatic cancer ranks 14th in the global cancer incidence rate and 7th in the global cancer mortality [1]. Approximately 495,733 new cases of pancreatic cancer are diagnosed each year worldwide and 466,003 deaths [1]. The incidence rate is almost the same as the death rate, which profoundly reflects the malignancy of pancreatic cancer. With the development of medical technology, there are many treatments for pancreatic cancer (PC), such as surgery, chemotherapy, immunotherapy, targeted therapy, radio frequency, HAIFU, and microbial therapy. However, the overall survival rate is only 9% [2]. Surgical treatment is considered to be the only way to cure PC., but the 5-year survival rate of patients receiving surgical treatment is only 15–25% [3]. Early identification of pancreatic cancer risk factors for intervention has become an essential means to reduce the incidence rate of pancreatic cancer. Current research shows that smoking, drinking, obesity, diabetes, pancreatitis, and pancreatic cancer family history are high-risk factors for pancreatic cancer [4].

The relationship between diabetes and pancreatic cancer is particularly complex. Although there is disagreement on the relationship between the duration of diabetes and the risk of pancreatic cancer, almost all studies show that the risk of pancreatic cancer in diabetes patients is significantly higher [5–7]. Clarifying the relationship between antidiabetic drugs and the incidence rate of pancreatic cancer has become a hot spot in clinical practice.

Metformin is the first-line drug of type 2 diabetes mellitus (DM), and its role in reducing the mortality of patients with pancreatic cancer is widely recognized [8, 9]. Specifically, compared with other drugs or no use of metformin, the overall survival period and 5-year survival rate of patients with pancreatic cancer treated with metformin significantly increased [10, 11]. However, its relationship with the incidence rate of pancreatic cancer has not yet been unified. Therefore, we conducted a more detailed and rigorous meta-analysis to clarify the relationship between the use of metformin in diabetes patients and the risk of pancreatic cancer.

## Materials and methods

### Guidelines

This paper is based on the Preferred Reporting Items for Systematic Reviews and Meta-Analyses (PRISMA). The agreement of this overview has been published in PROSPERO (Registration No: CRD42022359987).

### Retrieval strategy

From the beginning of the database construction to August 31, 2022, We performed an electronic search on PubMed, Embase, Web of Science, Cochrane Library, clinicalrials.gov, and China National Knowledge Infrastructure (CNKI) databases, using the keywords "metformin" OR "biguanide" OR "dimethyl biguanide" AND "pancreatic cancer" OR "pancreatic tumor" in "Title/Abstract", with no language restriction. All the studies retrieved were independently screened by two authors (Jian Hu and Hong-Dan Fan). We will consult with a third person(Qing-Song Mao) if there are different opinions in the literature screening process. To include sufficiently accurate literature, we also searched and screened the references included in the literature.

### Inclusion and exclusion criteria

The inclusive criteria were as follows: (1) case–control or cohort study; (3) reporting or including studies on the association between metformin use and pancreatic cancer risk; (4) reporting the Relative Risk (RR), Hazard Ratio (HR) or Odds Ratio (OR) and 95% confidence interval (CI) of pancreatic cancer, or providing data that we can calculate them.

The exclusion criteria were as follows: (1) cross-sectional studies; (2) duplicated studies; (3) preclinical studies (such as in vivo studies, primary studies, and animal studies); (4) abstracts, case reports, reviews, conferences, letters, and books; (5) only showing the relationship between metformin and pancreatic cancer mortality; (6) no full-text studies; (7) contrast agent containing metformin; (8) lacking necessary data.

### Data collection

Two investigators (Jian Hu and Hong-Dan Fan) independently extracted and then checked the extracted data by a third party (Qing-Song Mao). For each study, we recorded the following information: the first author, publication year, publication region/country, study design, basic characteristics (including baseline age, average age, and male proportion), the time of diagnosis of diabetes in the study population, sample size, study period, outcome indicators (including adjusted OR value and 95% CI), adjusted confounding factors and contrast agent. If there is no adjusted OR value and 95% CI, the crude OR value and 95% CI will be extracted. Suppose there are multiple groups (multiple control groups or test groups) in the literature that all meet the inclusion criteria. In that case, we extract or calculate the sample size data of each group and use the method of merging multiple groups of sample size into a new group to calculate the OR value and 95% CI [12]. Since the incidence rate of pancreatic cancer is low (less than 5%), the RR and HR values can be equated with OR values.

### Quality evaluation

This analysis uses the Newcastle Ottawa Scale (NOS) [13] to evaluate the method quality of the included studies. The score of NOS ranges from 0 to 9. We define studies with ≥ 7 points as high-quality studies in this analysis.

### Statistical methods

STATA MP 17.0 is adopted for all statistical analyses in this paper. The heterogeneity between studies was investigated by the Q test and measured by I^2^ statistics. If the I^2^ values exceeded 25%, 50%, and 75% respectively, it represented low, medium, and high heterogeneity [14]. When the I^2^ value is greater than 50%, the random effect model is used; otherwise, the fixed effect model is used. We conducted sensitivity analysis by excluding each study or some studies that may affect the stability of the study results and conducted subgroup analysis and single factor meta-regression analysis on some characteristics of the included studies. We assessed publication bias by visual funnel plots and the Egger regression asymmetry test. Unless otherwise stated, the statistical significance level was set at P < 0.05 under a double-sided test.

## Results

### Search process and results

**Fig.1:**
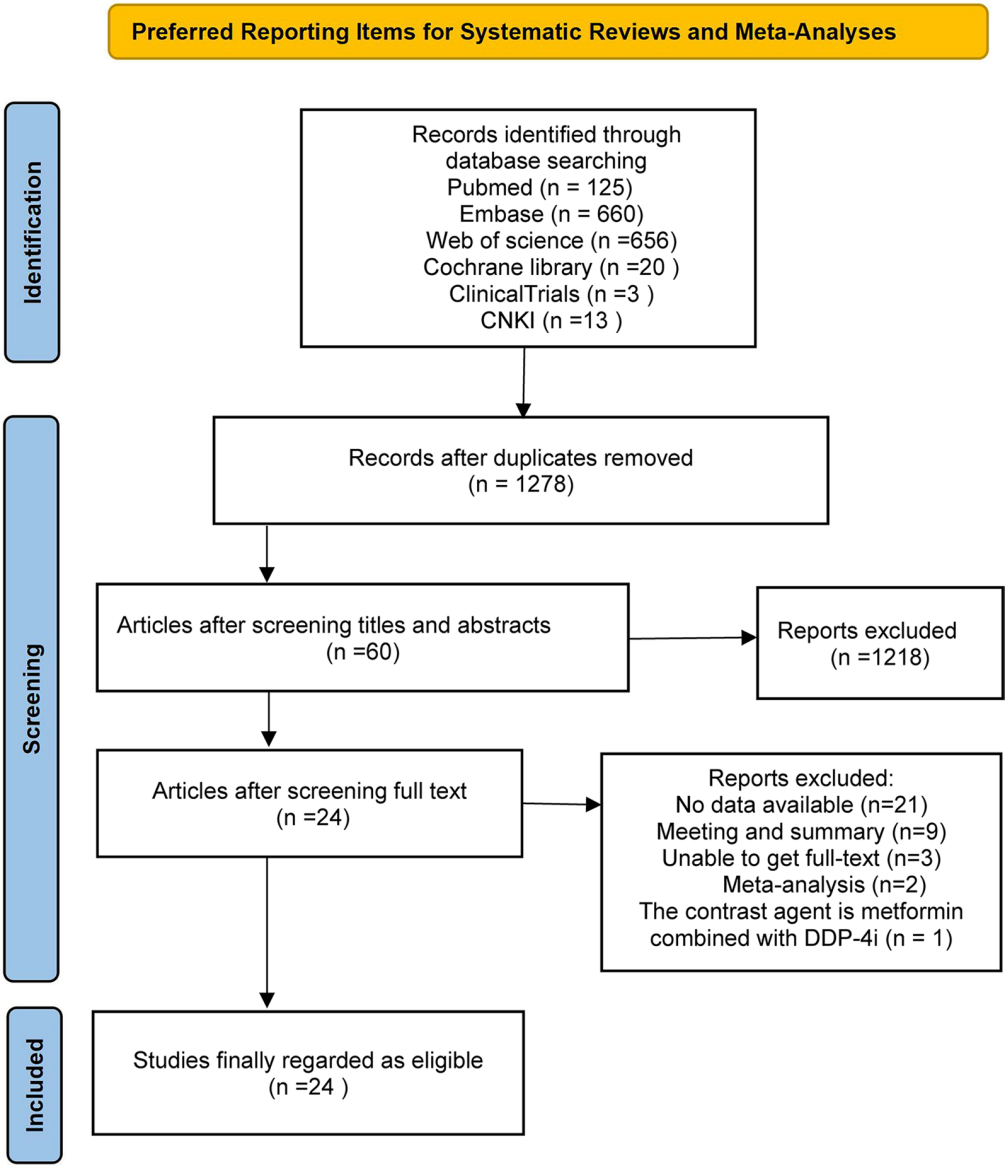
Flow diagram of study selection

Through the search of the above databases, we have preliminarily obtained 1477 articles that may be relevant. After importing the received articles into Note-Express, we found 199 duplicate articles. After reading the title and abstract, we excluded 1218 articles irrelevant to the study. Then, the remaining 60 articles were reviewed in full text, and 36 studies were excluded again. Among them, 21 studies had no available data, 9 were conferences or abstracts, three were unable to obtain the full text, 2 were meta-analyses or reviews, and one was treated with metformin combined with dipeptidyl peptidase-4 inhibitors (DPP-4i) as the contrast agent. Finally, the remaining 24 studies that met the inclusion criteria were analyzed. The retrieval and filtering process is shown in Fig. 1.

### Research characteristics

We included a total of 24 articles [15–38] (29 studies are included because some studies have multiple control groups or test groups), including 18 cohort studies and six case–control studies involving more than 2.28 million people. Their basic characteristics are shown in Table 1. Among the 24 articles, ten were conducted in Asia (seven [19, 20, 24–26, 31, 35] in China and three [22, 30, 36] in South Korea), and the remaining 14 were conducted in no-Asia (six [16, 18, 27, 29, 32, 37] in Britain, four [15, 23, 34, 38] in the United States, two [17, 28] in the Netherlands, 1 [33] in Italy and 1 [21] in Europe). Only two studies [24, 26] are of low quality. Four articles [19, 27, 35, 37] reported that many studies met the inclusion criteria, and the above methods were used to merge the study groups. All selected studies reported the results between the use of metformin and the risk of pancreatic cancer, but the reference group drugs they designed were not identical. The results of 13 studies [15, 18, 20–26, 28, 33–35] were not statistically significant. Eight studies [17, 19, 26, 27, 30, 31, 37, 38] reported that metformin significantly reduced the risk of pancreatic cancer, and three studies [29, 32, 36] reported an increase in the risk of pancreatic cancer.

**Table 1:** Characteristics of the included studies

First author, year	Country	Study design	Control origin	Contrast agent	No.of cases	No.of control	OR (95%CI)	Baseline age (year**)**	Mean age (years) (case/control)	Percentage of males (case/control)	Study period	Adjusting variables	New diabetes	NOS scores
Ruiter 2012	Netherlands	Cohort	Population	Sulfonylureas	52,689	32,591	0.73(0.66–0.80)	≥ 18	61.8/65.6	46.4/48.2	1998–2008	Age, gender, hypoglycemic agent duration, other drugs use, previous hospitalization	No	9
Bodmer 2011	Britain	Case–control	Population	No metformin	2763	16,578	0.83(0.57–1.21)	> 0	NA	46.2/46.2	1995–2009	BMI, smoking, drinking and the course of diabetes, congestive heart failure, ischemic heart disease, ischemic or hemorrhagic heart disease, transient ischemic attack, arterial hypertension and dyslipidemia, aspirin, other NSAIDs, statins or estrogen use	No	9
Sung 2020(a)^A^	Hong Kong, China	Cohort	Hospital	No metformin and aspirin	11,365	277,932	1.45(0.83–2.53)	≥ 18	NA	46.5/53.3	2000–2004	Age, gender, comorbidities, and baseline medications (including histamine 2 receptor antagonists (H2 antagonists), statins, nonsteroidal anti-inflammatory drugs (NSAIDs), and anticoagulants	No	9
Sung 2020(b)^A^					6630	277,932	0.58(0.20–1.65)		NA	52.8/53.3			No	9
Zhao 2022	China	Cohort	Population	Sulfonylureas	16,982	19,285	1.01(0.51–1.98)	≥ 18	58.1/61	53.2/51.0	2009–2020	Age, gender, education level, duration of smoking, drinking, T2DM, blood glucose level, blood lipid level and blood pressure, Charlson's complication index, BMI, and utilization rate of medical care; Sulfonylurea and metformin removal(α- Glucosidase inhibitors, thiazolidinediones, dipeptidyl peptidase 4 inhibitors, Grinnerd, and insulin), commonly used drugs for cardiovascular diseases (diuretics, β- Blockers, calcium channel blockers, angiotensin-converting enzyme inhibitors (ACEI), angiotensin receptor blockers (ARB) and aspirin), commonly used antibiotics (penicillins, cephalosporins, macrolides, quinolones, and other antibiotics), statins and proton pump inhibitors (PPIs)	Yes	9
Valente 2017	Europe	Case–control	Hospital	No metformin	164	529	1.35(0.68–2.66)	NA	59.6/59.5	51.0/51.0	2013–2015	Smoking, drinking, height and weight, body mass index (BMI), chronic pancreatitis, acute pancreatitis, peptic ulcer disease, biliary calculus and previous surgical history, gender, age, and inclusion in the center	No	7
Oh 2020	Korea	Cohort	Population	No metformin	19,546	19,546	0.88 (0.70–1.11)	≥ 18	60.5/60.3	53.0/52.6	2011–2015	Gender, socio-economic information (income level and residence in 2010), complications (hypertension, coronary artery disease, cerebrovascular disease, psychological and behavioral disorders, musculoskeletal diseases, chronic kidney disease, dyslipidemia, anemia, chronic obstructive pulmonary disease, arrhythmia, and liver cirrhosis); received surgery in 2010; and total hospital stay in 2010	No	9
Murff 2018	America	Cohort	Population	Sulfonylureas	42,217	42,217	0.85(0.57–1.27)	≥ 18	66.2/65.4	97.2/97.2	2001–2008	Age, gender, race (white, black, other), cohort entry date, body mass index, blood pressure, glomerular filtration rate, hemoglobin A1c (HbA1c), low-density lipoprotein level, smoking status, drug selection (statins, aspirin, antihypertensive drugs, anticoagulants, antiarrhythmic drugs, diuretics, antipsychotics, glucocorticoids), Times of medication and outpatient visits for comorbid diseases (cardiovascular disease, serious mental disease, heart valve disease, arrhythmia, Parkinson's disease, chronic obstructive pulmonary disease, liver disease)	No	9
Tsilidis 2014	Britain	Cohort	Population	Sulfonylureas	51,484	18,264	0.70(0.45–1.07)	≥ 35	61.1/65.3	56.1/57.9	1987–2010	Age, gender, body mass index, smoking. Alcohol consumption, aspirin or nonsteroidal anti-inflammatory drugs (NSAIDs), statins, and exogenous hormones	No	9
Wang 2013	Taiwan, China	Case–control	Population	No metformin	2158	8609	1.14(0.68–1.91)	N.A	NA	NA	1998–2009	Age, gender, and occupation	Yes	5
Liao 2012	Taiwan, China	Cohort	Population	No metformin	42,754	7049	0.85(0.39–1.89)	≥ 20	NA	NA	1998–2007	Age, gender, chronic pancreatitis, hepatitis C infection, gallstones	Yes	8
Oliveria 2008	America	Cohort	Population	No metformin	N.A	NA	1.26(0.80–1.99)	≥ 18	NA	NA	2000–2004	Age, gender, partial gastrectomy, chronic pancreatitis, deep venous thrombosis, dermatomyositis/polymyositis, alcoholism, hepatitis B/C, history of polyps	No	9
Tseng 2018	Taiwan, China	Cohort	Population	No metformin	12,616	12,616	0.49(0.25–0.96)	NA	NA	NA	1999–2005	Age, gender, occupation, residential area, hypertension, dyslipidemia, obesity, kidney disease, eye disease, stroke, ischemic heart disease; peripheral artery disease; chronic obstructive pulmonary disease, tobacco abuse; history of Helicobacter pylori infection; drugs (insulin, sulfonylurea, metronidazole, acarbose, rosiglitazone, pioglitazone, and angiotensin-converting enzyme inhibitors/angiotensin receptor blockers, calcium channel blockers, statins, fibrin, and aspirin)	Yes	6
Currie 2009(a)^B^	Britain	Cohort	Population	Sulfonylureas	31,421	7439	0.20(0.11–0.36)	≥ 40	58.6/70.0	51.1/54.9	2000-mid2000	Age, gender, systolic blood pressure, total cholesterol, weight, weight change, BMI, smoking status, baseline general incidence rate, previous major vascular disease (LVD), retinopathy, kidney damage, glycosylated hemoglobin, and previous solid tumor records	No	9
Currie 2009(b)^B^				Insulin	31,421	10,067	0.22(0.12–0.38)		58.6/63.7	51.1/55.4			No	9
De 2017	Netherlands	Cohort	Population	No metformin	37,215	19,899	1.11(0.72–1.71)	≥ 30	63.5/67.0	48.8/47	1998–2011	Age, duration of diabetes (time since NIAID dispensing was first recorded), other drugs (statins, aspirin, nonaspirin nonsteroidal anti-inflammatory drugs (NSAIDs), proton pump inhibitors, bisphosphonates, tamoxifen, oral contraceptives, and insulin)	No	9
Farmer 2019	Britain	Cohort	Population	No use of any medicine	6105	49,524	3.11(1.24, 7.76)	≥ 30	57.6/62.2	58.9/56.1	1990–2014	Age, gender, smoking status and alcohol status, year of onset of diabetes, HbA1c, BMI, previous year's use of other drugs (NSAIDs, statins, antihypertensive drugs), chronic kidney disease (CKD), and cardiovascular disease (CVD) history	No	9
Lee 2018	Korea	Cohort	Population	No metformin	688,656	277,797	0.86(0.77–0.96)	≥ 30	NA	NA	2009–2012	Age, gender, chronic pancreatitis, acute pancreatitis, hepatitis B, hepatitis C, biliary disease, alcoholism, NAFLD, lowest quartile income, place of residence, and number of ADMs with different exposure	Yes	9
Lee 2011	Taiwan, China	Cohort	Population	No metformin	11,212	4194	0.15(0.03–0.79)	≥ 20	NA	NA	2000–2007	Age, gender, another oral antidiabetic agent, CCI score, duration of metformin exposure	Yes	9
Lu 2015	Britain	Case–control	Population	No metformin	175	856	1.50(1.07–2.09)	≥ 20	NA	NA	1996–2010	Age, gender, BMI, smoking, drinking; Townsend deprivation index, and diabetes	Yes	8
Vicentini 2018	Italy	Cohort	Population	No use of any medicine(Dietary treatment)	7460	4060	1.51(0.59–3.89)	≥ 20	NA	NA	2009–2012	Gender, age, nationality, and time after diagnosis of diabetes	No	8
Walker 2015	America	Case–control	Hospital	No metformin	81	89	1.01(0.61–1.68)	≥ 21	NA	53/48.3	2006–2011	Age, gender, race, BMI, history of pancreatitis, alcohol, smoking, P.C. family history, other diabetes drugs, diabetes duration	No	9
You 2020	Korea	Cohort	Population	No metformin	131,877	131,877	1.34(1.21–1.48)	> 0	60.7/60.9	49.9/50.9	2005–2014	Age, gender, economic status, and residential area	Yes	9
Hsieh 2012(a)^C^	Taiwan, China	Cohort	Population	Sulfonylureas	3963	6072	0.63(0.28–1.42)	≥ 20	NA	NA	2000–2008	Age, gender	No	8
Hsieh 2012(b)^C^				Insulin	3963	751	1.44(0.18–11.5)	≥ 20	NA	NA			No	8
Van 2011(a)^D^	Britain	Cohort	Population	Sulfonylureas	109,708	68,029	0.60(0.52–0.70)	> 40	63.0/65.0	56.3/56.1	1997–2006	Age, gender, past years, social and economic status of small regions, smoking status, alcohol consumption, BMI, previous medical history (history of coronary heart disease, coronary artery reconstruction, hyperlipidemia, hypertension, peripheral vascular disease, renal damage, stable angina pectoris), previous medication (angiotensin II receptor blocker, antiplatelet β Receptor blockers, calcium channel blockers, diuretics, nitrates, NSAIDs, aspirin or statins)	No	9
Van 2011(b)^D^				Thiazolidinediones	109,708	31,372	1.16(0.91–1.48)	> 40	63.0/63.0	56.3/57.3			No	9
Van 2011(c)^D^				Insulin	109,708	23,005	0.46(0.38–0.56)	> 40	63.0/65.0	56.3/55.8			No	9
Li 2009	America	Case–control	Hospital	No metformin	255	106	0.38(0.22–0.69)	NA	NA	NA	2004–2008	Age, race, gender, smoking, alcohol, BMI, family history of cancer, diabetes duration, use of insulin	No	8

### Overall analysis

**Fig. 2:**
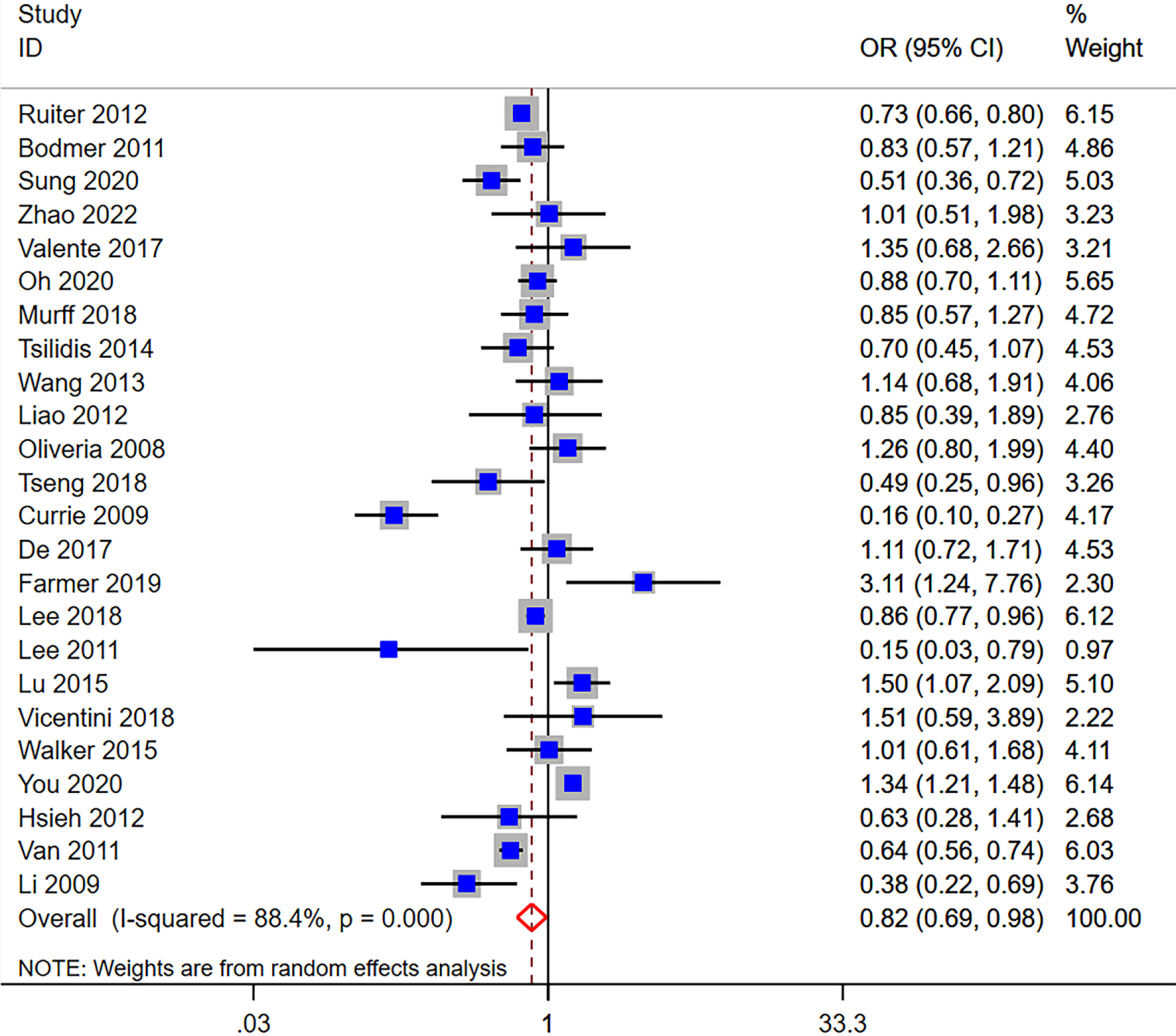
Forest plot of the association between metformin users and pancreatic cancer incidence

An overall analysis of 24 articles using the random effect model showed that compared with no use of metformin, the use of metformin could reduce the risk of pancreatic cancer in patients with type 2 diabetes (OR = 0.82, 95% CI (0.69, 0.98)), with significant heterogeneity (Q = 198.67, df = 14, p_Q_ = 0.000; I^2^ = 88.4%) (Fig. 2).

### Sensitivity analysis, subgroup analysis, and meta-regression

**Fig. 3:**
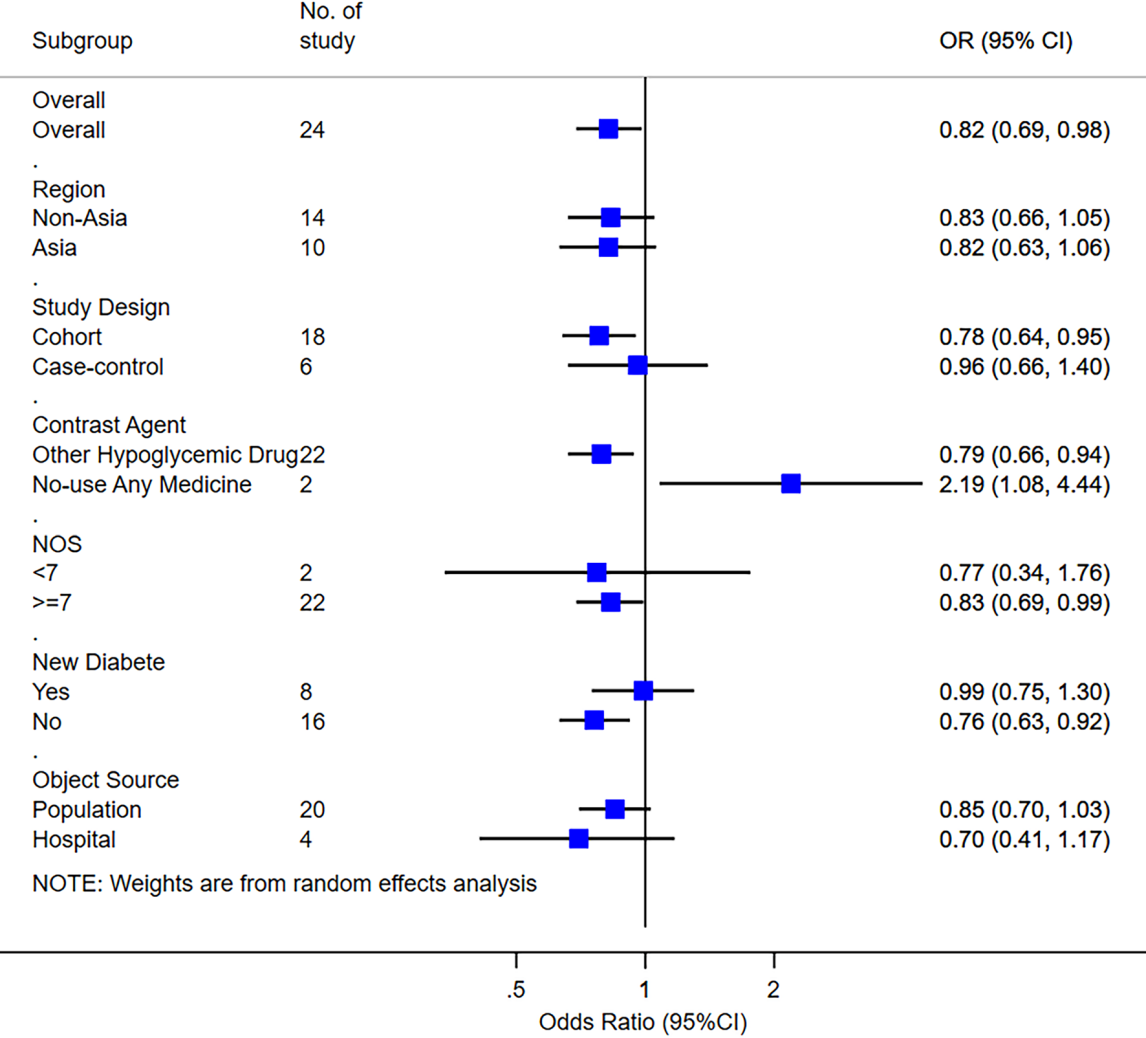
Summary of subgroup analysis results

To estimate the accuracy and robustness of the combined effect amount, we conducted a sensitivity analysis by excluding each study one by one and excluding some studies that may affect the research results (Table 2). There were four studies whose effect values came from the combination of multiple groups, but after all of them were excluded, the study showed no statistical significance (OR = 0.95, 95% CI (0.80, 1.12)). The sensitivity analysis result shows that the stability of the conclusion is acceptable. To further clarify the source of research heterogeneity, we selected the random effect model to conduct subgroup analysis and single-factor meta-regression analysis on the characteristics that may cause research heterogeneity, such as study area, study type, contrast agent, research quality, and diabetes status of study subjects. When the analysis is limited to a cohort study, high-quality study, no-newly-diagnosed diabetes population, and contrast agent, the research results are statistically significant (Fig. 3). Single factor meta-regression analysis found that the contrast agent may be one of the sources of heterogeneity (Table 3), which can explain 13.01% of the heterogeneity sources (p = 0.047, Adj R-square = 13.01%).

**Table 2:** Results of sensitivity analysis

Excluded study	Original OR and 95%CI	After excluding study		
		OR and 95%CI	I^2^	P_Q_值
Ruiter 2012	0.73 (0.66–0.80)	0.83 (0.68–1.01)	87.8%	0.000
Bodmer 2011	0.83 (0.57–1.21)	0.82 (0.69–0.98)	88.9%	0.000
Sung 2020	0.51 (0.36–0.72)	0.84 (0.71–1.01)	88.4%	0.000
Zhao 2022	1.01 (0.51–1.98)	0.82 (0.68–0.98)	88.9%	0.000
Valente 2017	1.35 (0.68–2.66)	0.83 (0.68–1.01)	88.8%	0.000
Oh 2020	0.88 (0.70–1.11)	0.82 (0.68–0.98)	88.9%	0.000
Murff 2018	0.85 (0.57–1.27)	0.82 (0.68–0.98)	88.9%	0.000
Tsilidis 2014	0.70 (0.45–1.07)	0.83 (0.69–0.99)	88.9%	0.000
Wang 2013	1.14 (0.68–1.91)	0.81 (0.68–0.97)	88.9%	0.000
Liao 2012	0.85 (0.39–1.89)	0.82 (0.69–0.98)	88.9%	0.000
Oliveria 2008	1.26 (0.80–1.99)	0.81 (0.67–0.96)	88.8%	0.000
Tseng 2018	0.49 (0.25–0.96)	0.84 (0.70–1.00)	88.8%	0.000
Currie 2009	0.16 (0.10–0.27)	0.88 (0.75–1.04)	85.7%	0.000
De 2017	1.11 (0.72–1.71)	0.81 (0.68–0.97)	88.9%	0.000
Farmer 2019	3.11 (1.24–7.76)	0.80 (0.67–0.95)	88.5%	0.000
Lee 2018	0.86 (0.77–0.96)	0.82 (0.67–1.00)	88.9%	0.000
Lee 2011	0.15 (0.03–0.79)	0.84 (0.70–1.00)	88.7%	0.000
Lu 2015	1.50 (1.07–2.09)	0.80 (0.67–0.95)	88.3%	0.000
Vicentini 2018	1.51 (0.59–3.89)	0.81 (0.68–0.97)	88.9%	0.000
Walker 2015	1.01 (0.61–1.68)	0.81 (0.68–0.98)	88.9%	0.000
You 2020	1.34 (1.21–1.48)	0.79 (0.68–0.93)	79.8%	0.000
Hsieh 2012	0.63 (0.28–1.41)	0.83 (0.69–0.99)	88.9%	0.000
Van 2011	0.64 (0.56–0.74)	0.83 (0.70–1.00)	87.6%	0.000
Li 2009	0.38 (0.22–0.69)	0.85 (0.71–1.01)	88.4%	0.000
Sung 2020 Currie 2009 Hsieh 2012 Van 2011	NA	0.95(0.80–1.12)	83.7%	0.000

**Table 3:** Single factor metaregression-analysis of different research characteristics

Covariates	Coefficient	SE	t	P >|t|	95% conf. interval	
Region	−0.079	0.249	−0.32	0.753	−0.596	0.437
Study design	0.208	0.275	0.76	0.457	−0.363	0.780
Contrast agent	−1.031	0.491	−2.10	0.047	−2.049	−0.013
NOS	0.068	0.453	0.15	0.883	-0.873	1.008
New diabetes	−0.184	0.258	−0.71	0.484	−0.719	0.352
Object source	−0.193	0.324	−0.60	0.558	−0.866	0.480

### Publication bias

**Fig. 4:**
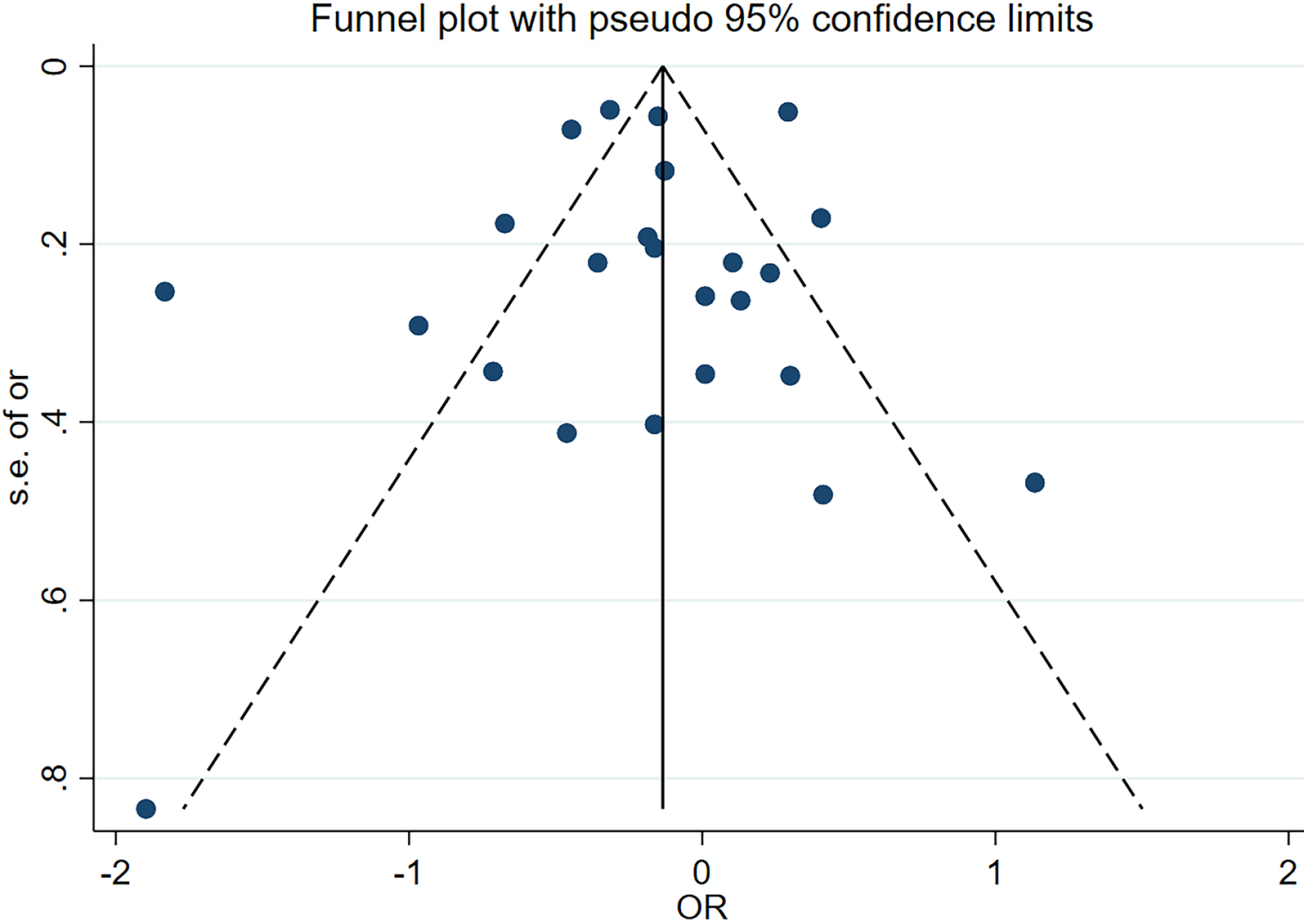
Funnel plot for publication bias in the studies

**Fig. 5:**
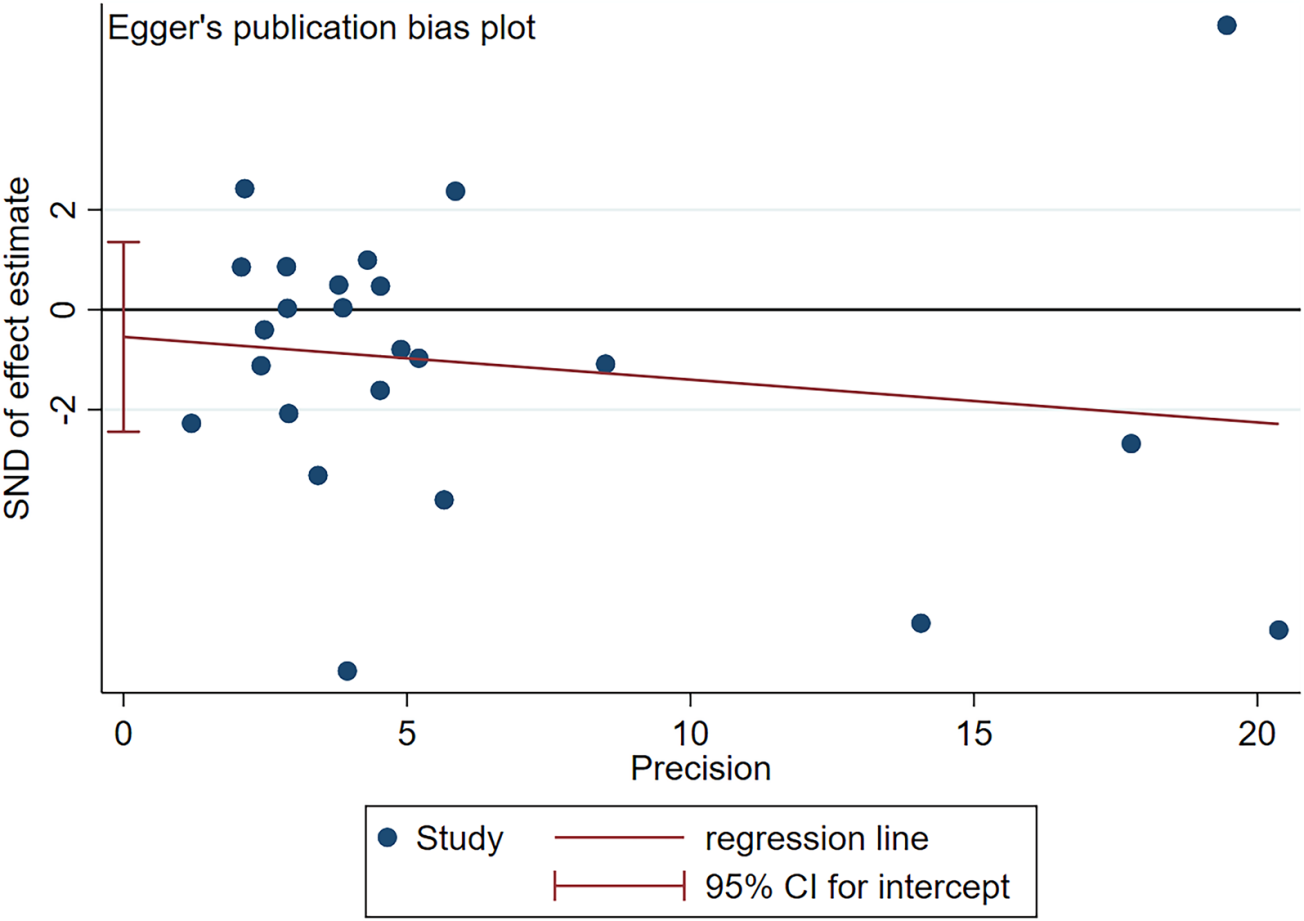
Egger's publication bias plot of the included studies

Finally, to evaluate the publication bias of the included studies, we intuitively evaluated the publication bias through the funnel chart (Fig. 4) and quantified it through the Egger regression. No significant publication bias was found (p = 0.445) (Fig. 5).

## Discussion

The epidemiology of cancer is constantly changing. As research showed [39], several aspects related to the epidemiology of liver cancer (such as etiology, clinical manifestations, treatment and treatment results) have changed dramatically from the previous ones, and the use of drugs may play an essential role in it. Meta-analysis has shown that statins have a specific chemopreventive effect on hepatocellular carcinoma [40]. A similar relationship may exist between some drugs and pancreatic cancer.

The mechanism and clinical research of diabetes increasing the risk of liver cancer have been studied in detail [41], but its relationship with pancreatic cancer still needs further investigation. Diabetes is a high-risk factor for pancreatic cancer and a possible consequence of pancreatic cancer [42]. To a certain extent, controlling diabetes mellitus can reduce the risk of developing pancreatic cancer. Metformin is one of the most commonly used oral hypoglycemic drugs in clinical practice, and its relationship with cancer has been widely studied. A study [43] investigating the impact of the use of metformin on the incidence rate or survival outcome of cancer showed that the use of metformin is related to reducing the incidence rate of pancreatic cancer and improving the overall survival of colorectal cancer, but there is no obvious evidence to show its correlation in other aspects. Some studies even believe that metformin is the first choice for the treatment of cancer patients with type 2 diabetes, because compared with other hypoglycemic drugs, the use of metformin can reduce the risk of death of cancer patients, especially in patients with pancreatic cancer, colorectal cancer and other cancers (except lung cancer, breast cancer cancer and prostate cancer [44]. Among them, studies on the survival rate or overall survival period of patients with metformin and pancreatic cancer are more frequent. Almost all studies show that patients with pancreatic cancer and diabetes can benefit from metformin [45, 46]. The anticancer effect of metformin is closely related to its powerful hypoglycemic effect. The effect of metformin on lowering blood glucose is carried out through the following ways: ① hepatic effect: improving hepatic insulin resistance, thus reducing hepatic glucose output, mainly reducing gluconeogenesis [47]; ② muscle effect: acting on skeletal muscle to increase insulin-stimulating glucose uptake and increase muscle AMPK activity and phosphorylation [48, 49]; ③ intestinal effects: changing intestinal microbial composition, changing hormone secretion (mainly growth and differentiation factor 15 and glucagon-like peptide-1), changing enterocyte glucose metabolism and delaying gastric emptying [50, 51].

This efficient hypoglycemic effect of metformin may contribute to reducing pancreatic carcinogenesis.

The current preclinical studies also confirmed the potential preventive effect of metformin on pancreatic cancer to some extent, although the evidence remains in animal (mouse) experiments. Metformin added in drinking water can prevent the pancreatic carcinogenesis induced by N-nitrosobis—(2-oxopropyl) amine in hamsters fed a high-fat diet [52]. In obese/pre-diabetes mice induced by diet, metformin reduced pancreatic tumor growth and mammalian target of rapamycin (mTOR) related signal transduction [53] (mTOR is a crucial complex involved in protein translation regulation). Metformin can prevent weight gain, liver steatosis, hyperlipoproteinemia, and hyperinsulinemia in KC (LSL-KrasG12D/ + ;p48-Cre) mice induced by high-fat and high-calorie diet. And it also can effectively prevent the progress of late PanINs and the development of KRAS( Kirsten Rat Sarcoma Viral Oncogene) driven pancreatic ductal adenocarcinoma promoted by diet-induced obesity [54]. Dong TS's [55] study showed that oral metformin could significantly change the regional microbiome of the duodenum and inhibit the development of PanIN lesions in the diet-induced obesity model of pancreatic cancer. Chen K [56] team found that the intake of metformin could delay the occurrence of pancreatic tumors through the study of KC mouse models, which showed that the percentage of early lesions and late mPanIN lesions (mPanIN2 and mPanIN3) decreased. In addition, metformin inhibits the tumorigenesis induced by chronic pancreatitis and may play a relevant role in reducing the pancreatic fibrosis induced by chronic pancreatitis. The combination of metformin and some drugs also reflects its role in cancer prevention to a certain extent. Metformin and rapamycin can inhibit pancreatic tumor growth in obese and pre-diabetes mice through common and different mechanisms [53]. It was proved that the combination of metformin and aspirin significantly inhibited tumor growth and downregulated the protein expression of Mcl-1 and Bcl-2 in tumors in the xenotransplantation mouse model [57], which has preventive significance for the occurrence of pancreatic cancer. The emergence of these mechanisms seems to indicate that metformin does play a role in reducing the incidence of pancreatic cancer.

However, as far as the published meta-analysis is concerned, its role is still uncertain. Wang Z [58], Yu X [59], and Zhang P [60] all showed that metformin is a protective factor for pancreatic cancer, which can reduce the incidence of pancreatic cancer by 37%, 36%, and 46%. However, Singh S [61] suggested no significant correlation between metformin and pancreatic cancer (OR = 0.76, 95% CI 0.57–1.03). A recent meta-analysis [62] on the relationship between metformin and the incidence of total cancer also showed that using metformin could reduce the risk of pancreatic cancer. According to the difference in the control group, the study was divided into the group that has never used metformin and the group that has used other anti-diabetes drugs (OR = 0.62, 95% CI (0.45,0.84)); OR = 0.57, 95%CI (0.35,0.93)).

Since there is no consensus on the role of metformin in the existing meta-analysis results, we conducted this meta-analysis involving 24 articles. In this analysis, more than 2.28 million people participated. The overall analysis of the study showed that the use of metformin was negatively correlated with the occurrence of pancreatic cancer (OR = 0.82, 95% CI (0.69, 0.98)), which was consistent with most previous studies. When subgroup analysis is conducted according to study quality, only the subgroup of the high-quality study shows that metformin is negatively related to the risk of pancreatic cancer, which may be due to the deviation of the research methodology of the low-quality study. When the subgroup analysis was carried out according to the status of diabetes of the study subject, only the subgroup of non-newly diagnosed diabetes suggested that metformin was negatively related to the risk of pancreatic cancer, which may be because the protective effect of metformin on pancreatic cancer needs a certain delay. When subgroup analysis is conducted according to the study design, metformin can reduce the risk of pancreatic cancer only in the cohort study subgroup, which may be caused by the relatively small sample size of the case–control study and the low statistical efficiency in the study. It is worth noting that when the contrast agent was sub-analyzed, the opposite results were obtained. Single-factor meta-regression showed that the contrast agent was one of the heterogeneities of the study. The overall sensitivity analysis indicated that the study was stable, and no significant publication bias was found through the funnel plot and Egger test.

Compared with the previous meta-analysis, our research has some advantages. Firstly, this paper has included 24 articles from many countries, including more than 2 million participants, with high study quality, enhancing the statistical power of the data analysis and providing more reliable estimates. Secondly, we explored the research heterogeneity through subgroup analysis and single-factor meta-regression. Fortunately, we found the source of some research heterogeneity. Finally, since the existing evidence shows a relationship between the duration of diabetes and the occurrence of pancreatic cancer, we conducted a subgroup analysis on whether the study subjects were newly diagnosed with diabetes and obtained inconsistent results. As far as we know, this is the first meta-analysis of this subgroup analysis. When researchers later conduct relevant research, it can remind them to consider the diabetes status of the subjects.

However, we must admit that this study has some limitations. First of all, the heterogeneity of the study is remarkable. Although we have carried out some subgroup analysis, sensitivity analysis, and meta-regression, we only found partial sources of heterogeneity. The rest of the heterogeneity may be attributed to retrospective studies, inconsistent adjustment of confounding factors, or inconsistent follow-up time. Second, although we think that the flushing period and lag period will significantly impact the research results due to the inability to extract relevant data in some studies, no further analysis can be conducted. Third, the contrast agents of all the studies included in the analysis differ. Most appear as "no metformin users", but the specific drugs they contain are unclear. Although we have conducted subgroup analysis, whether "no metformin users" includes "no drug users" is ambiguous, which may lead to errors and bias in the results. Fourth, part of the literature contains several studies. We calculated and combined the sample size to obtain data for analysis, which may be biased from the actual situation. Fifth, we have extracted risk estimates that reflect the maximum control of potential confounding factors. However, the results of adjustments based on specific confounding factors may be different from those based on standards.

## Conclusion

Metformin can reduce the risk of pancreatic cancer in patients with diabetes. Prospective research is needed to confirm our view in the future further.

## Data Availability

All data and materials from this study are presented within the manuscript.

## Abbreviations

PC: Pancreatic cancer
DM: Diabetes mellitus
PRISMA: Preferred Reporting Items for Systematic Reviews and Meta-Analyses
CNKI: China National Knowledge Infrastructure
NOS: Newcastle Ottawa Scale
DPP-4i: Dipeptidyl peptidase-4 inhibitors
mTOR: Mammalian target of rapamycin
KRAS: Kirsten Rat Sarcoma Viral Oncogene
KC: LSL-KrasG12D/ + ;p48-Cre
RR: Relative Risk
HR: Hazard Ratio
OR: Odds Ratio
